# P-909. Molecular Testing Performance for the Diagnosis of Cryptococcal Meningitis in a Tertiary Care Center

**DOI:** 10.1093/ofid/ofae631.1100

**Published:** 2025-01-29

**Authors:** Ruth Serrano Pinilla, Manuel Endo, Steven Dallas

**Affiliations:** UT Health San Antonio, San Antonio, Texas; UT health San Antonio, San Antonio, Texas; UT Health San Antonio, San Antonio, Texas

## Abstract

**Background:**

Cryptococcus is the most common cause of fungal meningitis worldwide and delayed diagnosis and treatment can be devastating. Diagnostic modalities include culture, antigen detection (CrAg), direct visualization in CSF and PCR. The reported sensitivity of the FilmArray® meningitis/encephalitis (ME) panel for cryptococcal meningitis compared to culture is 92-96%, and compared to CSF CrAg is 52-83%, with reported specificity of 99%. Lower CSF CrAg levels and lower quantitative growth in culture have been associated with false negative ME panel.

Characteristics of samples with false negative ME Panel

**Figure:**
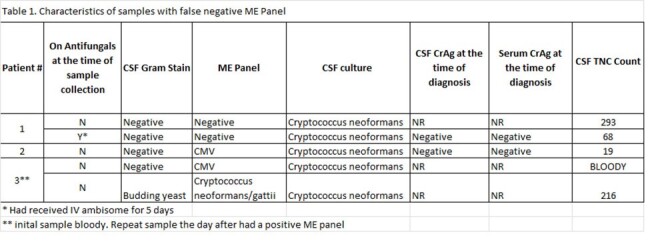

**Methods:**

After IRB approval was obtained, a retrospective chart review of all cases of cryptococcal meningitis established by CSF Culture, CSF CrAg or ME panel from June 2017-June 2020 in a tertiary center was performed.

**Results:**

*Cryptococcus spp*. was detected in 18 specimens, by either ME panel (14), CSF CrAg (12) or culture (18). These specimens were obtained from 16 patients. Their mean age was 46 years (24-73). 75% of patients were male. All the patients in this series were immunocompromised. 10 patients had HIV (PWH), half of them had CD4 counts < 40 cells/mm^3, and the mean CD4 count of the rest was 99 cells/mm^3 (41-163). Three patients had a prior solid organ transplant, one had liver cirrhosis and one had idiopathic CD4 lymphopenia. The mean opening pressure was 28.1 (16- >60 cm CSF). Compared to culture, the ME panel detected *Cryptococcus spp*. in 14/18 specimens. Of those, a total of 14 patients had a simultaneous CSF CrAG performed, 12 of those samples had a detectable CrAG, and ME panel detected the organism in all those samples. Both false negative CSF CrAG had a false negative ME Panel but were later diagnosed with a culture. There were 4 samples with false negative ME Panel. Their cultures were reported as “very light” qualitative growth. Two of them had a bloody sample and CMV detected in the ME panel, suggesting that contamination with large amount of blood can interfere with the performance of this test. This delayed treatment initiation by an average of 2 days.

**Conclusion:**

Although the ME Panel is an excellent tool to diagnose meningitis, the lack of detection of Cryptococcus sp. in the ME panel in the right clinical context should prompt careful clinical consideration to prevent delays in treatment initiation for patients at risk of this infection.

**Disclosures:**

**All Authors**: No reported disclosures

